# Draft Genome Sequences of *Leviviridae* RNA Phages EC and MB Recovered from San Francisco Wastewater

**DOI:** 10.1128/genomeA.00652-15

**Published:** 2015-06-25

**Authors:** Alexander L. Greninger, Joseph L. DeRisi

**Affiliations:** a Department of Biochemistry and Biophysics, UCSF, San Francisco, California, USA; b Howard Hughes Medical Institute, UCSF, San Francisco, California, USA

## Abstract

We report here the draft genome sequences of marine RNA phages EC and MB assembled from metagenomic sequencing of organisms in San Francisco wastewater. These phages showed moderate translated amino acid identity to other enterobacteria phages and appear to constitute novel members of the *Leviviridae* family.

## GENOME ANNOUNCEMENT

*Leviviridae* is a family of positive-stranded RNA bacteriophages consisting of two genera, *Allolevivirus* and *Levivirus*, which are each generally restricted to a single host genus of Gram-negative bacteria (1). Of note, the RNA-binding coat protein of the *Levivirus* MS2 phage has served as a tool for many basic science applications and as a model for the assembly of RNA viruses (2, 3). Interestingly, surveys performed in 1976 and 2006 of marine/coastal RNA virus communities found very few if any RNA phages (4).

While performing weekly metagenomic sequencing of organisms in San Francisco wastewater, we assembled 3,180-nucleotide (EC) and 3,925-nucleotide (MB) contigs at 849× and 229× coverage, respectively, which aligned by BLASTx to enterobacterial phages Hgal1, M11, and Qbeta. Each contig comprised a typical *Leviviridae* genome organization, consisting of three open reading frames (ORFs) encoding maturation, coat, and replicase proteins, with phage EC likely truncated by ~600 nucleotides at the 3′ end. However, neither phage encoded a readily apparent lysis or readthrough protein. Phage EC appears to possess ORFs that encode both coat and replicase proteins but lacks any protein with alignment to known lysis proteins of *Levivirus*. Phage MB also appears to lack a recognizable lysis protein.

The predicted replicase protein of phage MB demonstrated 39% amino acid identity to *Caulobacter* phage phiCb5 over the entirety of the replicase protein, while the closest replicase to phage EC demonstrated 42% amino acid identity to enterobacterial phage Hgal1. Both replicases demonstrated <38% identity to members of *Leviviridae* already placed in a genus, which is equivalent to amino acid identity between *Allolevivirus* and *Levivirus* genera, consistent with these two viruses forming two novel genera within *Leviviridae*. The predicted maturation proteins aligned with 32% amino acid identity to enterobacterial phage BZ13 (EC) and 28% to *Caulobacter* phage phiCb5 (MB). The predicted phage EC coat protein aligned with 32% amino acid identity to enterobacterial phage C-1 INW-2012, while the predicted phage MB coat protein failed to demonstrate significant amino acid alignments to any phage proteins but did significantly align by HHPred to *Caulobacter* phiCb5 virus-like particle (5, 6).

The sample that yielded these draft genome sequences was prepared from 1 liter of wastewater that was concentrated to <5 ml of particles between the size of 0.22 μm and 300 kDa using Millipore Pellicon XL 300-kDa filters and 0.22-μm spin columns. Nucleic acid was extracted using the Zymo viral DNA/RNA kit, and half of the recovered nucleic acid was treated with DNase. The phage genomes were discovered and assembled using PRICE version 1.0, Geneious version 8.0 Assembler, and SURPI version 1.0 from a total of 15,719,690 paired-end 65-bp reads sequenced on an Illumina GAIIx split between these DNAsed and untreated nucleic acid preparations (7, 8).

### Nucleotide sequence accession numbers.

The GenBank accession numbers for marine RNA phages EC and MB are KF616862 and KF510034, respectively.
